# Managing the Fight against Onchocerciasis in Africa: APOC Experience

**DOI:** 10.1371/journal.pntd.0003542

**Published:** 2015-05-14

**Authors:** Grace Fobi, Laurent Yameogo, Mounkaila Noma, Yaovi Aholou, Joseph B. Koroma, Honorat M. Zouré, Tony Ukety, Paul-Samson Lusamba-Dikassa, Chris Mwikisa, Daniel A. Boakye, Jean-Baptist Roungou

**Affiliations:** 1 World Health Organization/African Programme for Onchocerciasis Control (WHO/APOC), Ouagadougou, Burkina Faso; 2 World Health Organization (WHO), Geneva, Switzerland; University of Cambridge, UNITED KINGDOM

## Introduction

Due to the socioeconomic impact of human onchocerciasis (commonly referred to as river blindness) in West Africa, the Onchocerciasis Control Programme in the Volta River Basin (OCP) was instituted [1]. This initial programme started in 1975 and covered seven West African countries: Benin, Burkina Faso, Cote d’Ivoire, Ghana, Mali, Niger, and Togo. However, later evidence indicated that endemic areas outside the initial area posed a threat to the achievement of the OCP and, hence, the Programme was extended southward and westward to include four additional countries, bringing the total number of countries covered by OCP to eleven. The formal name was then changed to the Onchocerciasis Control Programme in West Africa, retaining the acronym OCP.

**Fig 1:**
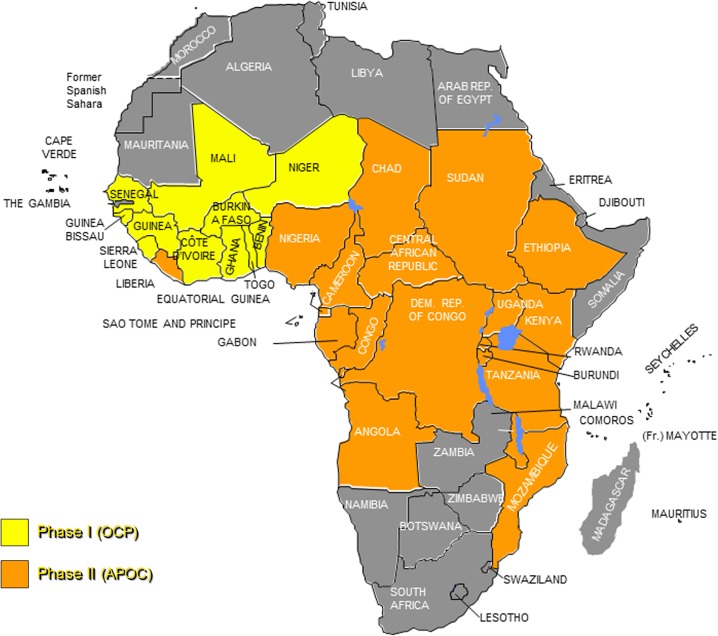
Onchocerciasis-endemic countries in Africa, showing countries covered by the OCP and initially by APOC. Map from 2010. Note that South Sudan gained independence in 2011, becoming the 20th APOC country.

OCP used aerial larviciding as its principle strategy to control the vectors of human onchocerciasis, members of the *Simulium damnosum* complex, in the absence of a safe drug for mass treatment against the parasites [2]. Efforts to control onchocerciasis evolved in 1987 when ivermectin was donated to kill the juvenile worms that cause the various symptoms associated with the disease. As a result of the donation, OCP instituted a new strategy of chemotherapy in combination with vector control. In the 11 countries covered by OCP, this two-prong approach led to the virtual elimination of onchocerciasis as a public health problem and as an obstacle to socioeconomic development. The availability of a donated drug effective against the parasite and safe for mass drug administration, coupled with evidence that other pathological effects of onchocerciasis were equally important socioeconomic threats, led to the decision that onchocerciasis should be controlled in all endemic countries in Africa (Fig 1).

The African Programme for Onchocerciasis Control (APOC) was launched in December 1995. In order to reach its objective of onchocerciasis control in all endemic countries in sub-Saharan Africa, the Programme used Rapid Epidemiological Mapping of Onchocerciasis (REMO) [3] to delineate areas of mesoendemicity and hyperendemicity and to estimate the population at high risk of contracting onchocerciasis. Countries included in the APOC program were: Angola, Burundi, Cameroon, Central African Republic, Chad, Congo, Democratic Republic of Congo, Equatorial Guinea, Ethiopia, Gabon, Kenya, Liberia, Malawi, Mozambique, Nigeria, Rwanda, South Sudan, Sudan, Uganda, and Tanzania. The exercise revealed that 102 million people in the Programme area were at risk and needed ivermectin treatment, while an estimated 37 million people were already infected with the disease [4]. In 1997, APOC adopted community-directed treatment with ivermectin (CDTi) as its core strategy [5–7]. Following CDTi introduction and implementation, coverage and compliance with ivermectin steadily improved—the number of persons benefiting from ivermectin treatment increased from 1.5 million in 1997 to 75.8 million in 2010 and to 100.79 million in 2013. CDTi ensured a sustainable method to deliver ivermectin and also strengthened health systems.

Long-term impact assessments of APOC operations revealed a decrease in the number of persons infected from 37.9 million in 1995 to 15.1 million in 2011. An estimated 9.5 million cases of severe itching were prevented, 400,000 persons were protected from low vision, and 200,000 persons were protected from blindness. In most advanced APOC projects, the prevalence of infection is already close to zero. The operations of APOC prevented 8.9 million disability-adjusted life years (DALYs) from 2005–2010, with another estimated 10.1 million averted from 2011–2015 [8]. Through co-implementation activities, APOC has also averted an additional 1 million DALYs for other targeted diseases such as ascariasis, trichuriasis, hookworm, lymphatic filariasis, strongyloidiasis, and epidermal parasitic skin diseases over the duration of the Programme [9].

Research now shows that ivermectin treatment can not only control, but in many areas (Mali, Senegal, Uganda, and Nigeria), eliminate river blindness infection and interrupt transmission [10–12]. In 2009, taking into account the feasibility of the elimination of onchocerciasis infection and interruption of its transmission with ivermectin mass treatment alone [10], the Joint Action Forum (JAF), the governing body of APOC (described below), directed the Programme to shift from control to elimination of onchocerciasis. In 2010, the third midterm evaluation of APOC advised the JAF that it would be premature to close the Programme in 2015 given the perspective of onchocerciasis elimination. Thus, in 2011, JAF reaffirmed its endorsement for the Programme to pursue the elimination of onchocerciasis in Africa as well as co-implementation of preventive chemotherapy interventions for other selected neglected tropical diseases (NTDs) in the context of increased support to community-level health systems strengthening. The other preventable NTDs susceptible to mass drug administration include lymphatic filariasis (elephantiasis), trachoma, schistosomiasis (bilharzia), and soil-transmitted helminths, which include roundworm (ascariasis), whipworm (trichuris), and hookworm.

## Participative Governance

The Programme is a unique partnership between the affected communities, governments, bilateral and multilateral agencies, foundations, non-governmental development organizations (NGDOs), the scientific community, and the private sector. The partnership is built on a legal agreement called the “Memorandum of the African Programme for Onchocerciasis Control” [13]. The APOC Secretariat is responsible for initiating the budget process, taking the lead in preparing a multi-year plan of action for APOC and using this to develop an indicative budget to implement the multi-year plan. APOC is governed by the JAF, consisting of representatives of (a) the participating countries; (b) the contributing development partners; (c) the sponsoring agencies; (d) members of the NGDO Coordination Group; (e) Merck & Co., Inc. representing the private sector as the donor of ivermectin; (f) intergovernmental regional or sub-regional organizations; and (g) other invited entities. The JAF decides on the overall policy and strategy of APOC, assesses progress review, approves the APOC Plan of Action and Budget, and assesses global financing requirements of the Programme. The JAF meets annually and is usually hosted alternatively by a participating country and a donor country.

The Committee of Sponsoring Agencies (CSA), comprising representatives from sponsoring agencies, NGDO Coordination Group, Merck & Co., Inc., and the Mectizan Donation Program (MDP), works closely with the APOC Secretariat. CSA makes interim decisions on behalf of JAF when required, follows closely the financial situation of APOC, and scrutinizes documentation for the JAF. WHO is the executing agency within this partnership and the World Bank is the fiscal agent of the Programme [13]. The World Bank mobilizes donor contributions into the APOC Trust Fund and provides funds for APOC’s operations. Although APOC provides some contribution to the NGDO Coordination group through the Trust Fund, most of the funds from the NGDO group to countries are raised by the individual members of the group.

## Programme Management

APOC is one of the few programs that the WHO Regional Office for Africa implements directly. The WHO Regional Director for Africa ensures the overall guidance of APOC Secretariat, which is headed by the APOC Director. WHO Headquarters provides administrative and technical as well as operational research support. APOC maintains close collaboration with WHO offices of all participating countries and with National Onchocerciasis Task Forces (described below) in the implementation and monitoring of CDTi projects.

The Secretariat of the Programme is based in Ouagadougou, Burkina Faso, and runs two technical units: Sustainable Drug Distribution Unit and Epidemiology and Vector Elimination Unit. Administrative support is provided through the Director’s Office Coordination Unit. APOC currently supports onchocerciasis control and elimination activities in 31 African countries, including the 19 original signatories of the Memorandum, South Sudan, and the 11 ex-OCP participating countries. These countries included: Angola, Benin, Burkina Faso, Burundi, Cameroon, Central African Republic, Chad, Congo, Côte d’Ivoire, Democratic Republic of Congo, Equatorial Guinea, Ethiopia, Gabon, Ghana, Guinea, Guinea Bissau, Kenya, Liberia, Malawi, Mali, Mozambique, Niger, Nigeria, Rwanda, Senegal, Sierra Leone, South Sudan, Sudan, Tanzania, Togo, and Uganda. The main activities implemented by the Secretariat include the design, execution, monitoring, and evaluation of community-directed ivermectin distribution projects, as well as mapping of the disease, capacity building for countries, and provision of technical guidance in efforts to control and eliminate river blindness in Africa.

## Sustaining Partnership

APOC’s broad partnership includes the poor in programmatic decision-making. This partnership involves over 146,000 local communities, African endemic countries, donor countries and institutions, over 16 NGDOs, Merck & Co. Inc., research institutions, and programs such as the WHO Special Programme for Research and Training in Tropical Diseases (TDR), as well as research institutions within onchocerciasis-endemic countries.

### Engaging Communities

The CDTi strategy is built on a participation paradigm in which communities play an important role for planning, leading, and managing interventions that benefit their own health. In the CDTi context, a community is the lowest autonomous unit whose members are linked to one traditional or political head and share resources in common. CDTi focuses on empowering communities to take responsibility for ivermectin delivery by deciding how, when, and by whom the ivermectin treatment should be administered. This strategy seeks to empower the people who are the most affected to take specific roles, responsibilities, and critical decisions for interventions that address their needs.

CDTi, using a bottom-up approach, is a well-tested and highly cost-effective strategy that has extended the reach of essential interventions to those at the end of the road at a reasonably low cost. The success of CDTi, especially in remote areas of countries affected by conflict and war, has opened the doors to a number of other health care interventions that lend themselves to the community-directed intervention approach (CDI) [4,14]. There is evidence that at least four to five interventions could effectively be implemented through CDI with a boost in ivermectin coverage by 10% [15,16].

Empowered communities have contributed to improving the prevailing therapeutic coverage with ivermectin from 62.2% to at least 65% and geographical coverage to 100% [17]. CDTi/CDI is undertaken at the community level under the direction of the community itself. The health services and NGDOs introduce the concept of community ownership and role, whereby the community takes charge of the process.

Without community engagement in planning, designing, implementing, and monitoring, it is difficult for an external agent to identify the various social factors that will influence intervention implementation and service absorption within the village and to reach expected results. Using trained community-directed distributors (CDDs) selected by the community, APOC has been able to scale up treatment with ivermectin from 1.5 million in 1995 to over 75 million people in 2010. Engaging communities has yielded multiple health gains to remote communities, providing additional health interventions and commodities such as medicines for the control of other preventable NTDs, insecticide-treated bed nets for malaria control, and vitamin A supplementation.

### Engaging Governments and Other Stakeholders

All of the APOC participating countries have established a National Onchocerciasis Task Force (NOTF) composed of Ministry of Health (MoH) staff from relevant divisions, representatives of implementing NGDO partners, research institutions and representatives from other related Ministries (e.g., Ministry of Education). The National Onchocerciasis Control Programme (NOCP) serves as the Secretariat of the NOTF.

The NOTF has the responsibility of overseeing implementation of the onchocerciasis control efforts at the national level. The MoH has the critical role to create a favorable environment for all partners and enabling policies for community-directed interventions for the control and elimination of onchocerciasis and other NTDs targeted by preventive chemotherapy (PC-NTDs); ensuring entry of ivermectin and other NTD medicines in the country without imposing duty, tax, or other charges; as well as chairing and expanding the NOTF to include coordination of control and elimination of onchocerciasis and other PC-NTDs. The MoH also advocates for and mobilizes national financial contributions. Between 2010 and 2011, governments of 15 countries disbursed US$16,937,214 for CDTi-related equipment and salaries of health personnel of various CDTi implementation units. Those countries included: Angola, Burundi, Cameroun, Central African Republic, Chad, Congo, Democratic Republic of the Congo, Equatorial Guinea, Ethiopia, Liberia, Malawi, Sudan, Nigeria, Uganda and United Republic of Tanzania. These governments also disbursed US$3,012,750 to core CDTi activities alone [18]. However, in order to ensure elimination of onchocerciasis, the governments of participating countries need to increase financial and human resources for the following core CDTi activities: health education, sensitization advocacy and mobilization, training, distribution of ivermectin, supervision, monitoring, and reporting.

### Ensuring Medicine Availability

In 1987, Merck committed to donate Mectizan (ivermectin, MSD) for the treatment of onchocerciasis to all countries that need it for as long as necessary. In 1998, the donation was expanded to the treatment of lymphatic filariasis in the African countries where onchocerciasis and lymphatic filariasis are co-endemic. Medicine donation programs are critical as they cover a major technical and financial component of the control and elimination of onchocerciasis and other PC-NTDs. Sustaining the action of such programs is a key determinant for success in the fight against onchocerciasis and other NTDs. Between 1997 and 2011, the Mectizan Donation Programme (MDP) supplied 2,168,732,700 tablets to APOC participant countries. In 2011 alone, 352,594,500 (77%) tablets were distributed to APOC participant countries and 107,939,500 (23%) tablets were distributed to ex-OCP member countries for onchocerciasis or for integrated lymphatic filariasis and onchocerciasis control [19].

### Coordinating NGDO Actions

The WHO Programme for the Prevention of Blindness created the NGDO Coordination Group for Ivermectin Distribution in partnership with seven NGDOs in 1991, which later became known as the NGDO Coordination Group for Onchocerciasis Control at the launch of APOC in 1995. The membership of the Group increased to 12 NGDOs in order to address the need of expansion of the Programme in Cameroon, Nigeria, and Uganda. Since 2005, additional NGDOs have joined the Group as the result of increased momentum towards controlling NTDs. To date, the Group comprises 14 full members and three associate members. The full members include: Charitable Society for Social Welfare (CSSW), Christoffel-Blindenmission (CBM), Helen Keller International (HKI), IMA World Health, Lions Club International Foundation (SightFirst Program), Malaria Consortium, Mectizan Donation Program (MDP), Mission to Save the Helpless (MITOSATH), Organisation pour la Prévention de la Cécité (OPC), Schistosomiasis Control Initiative (SCI), SightSavers International, The Carter Center, United Front Against River Blindness (UFAR), and US Fund for UNICEF. The three associate members include: International Agency for the Prevention of Blindness (IAPB), Liverpool Centre for Neglected Tropical Diseases (CNTD), and Merck & Co, Inc. The NGDO Coordination Group for Onchocerciasis Control provides managerial, technical, and financial support to more than 80% of CDTi projects in participating countries. NGDOs also get involved in operational research activities. During the last few years, intra-country collaboration among members of the Group has allowed addressing the financial constraints faced by some of their members in order to sustain support to CDTi projects.

### CDTi Projects for Field Operations

APOC has delineated CDTi project areas using data obtained through REMO. Each CDTi project covers a delineated geographic area of an endemic country. The CDTi project approach allows for a phased introduction of CDTi in a country and focuses support on the early phases of CDTi development with the view of applying the lessons learned to the rest of the country. In 2010, treatment activities in 16 APOC participating countries covered 138,448 out of 144,837 (96%) endemic communities [19]. In total, 75.8 million people were treated with an average therapeutic coverage of 79.0% in countries with a stable security situation and 71.4% in post-conflict countries [19]. APOC presently supports 122 CDTi projects in 20 APOC participant countries and four ex-OCP member countries, Cote d’Ivoire, Ghana, Guinea Bissau, and Sierra Leone.

## Technical Oversight and Evaluation

The APOC Technical Consultative Committee (TCC) ensures the technical oversight of APOC operations. Its main function is to review new CDTi projects plans and budget, annual technical reports from CDTi projects and operational research proposals. TCC thus contributes to establishing a research agenda for APOC. Its recommendations are addressed to the Programme Director or, if required, to the CSA. The TCC members meet twice a year. The TCC comprises 12 members which are selected through various mechanisms. One of the 12 TCC members is a representative from MDP and is appointed by Merck & Co. Inc. Eleven members are scientists/experts appointed by the WHO Director-General based upon the recommendation of the CSA. Among those, three members are proposed by the NGDO Coordination Group for the consideration of the CSA. The other eight members are suggested by APOC management to the CSA for their consideration. TCC members appointed by the Executing Agency hold membership for three years renewable for a maximum of another three years, on a staggered basis. However, since MDP oversees the donation of the drug ivermectin, the MDP representative has permanent tenure on the TCC.

The review function of TCC has been partly devolved to some countries, including Cameroon, Nigeria, and Uganda, where Technical Review Committees (TRC) have been established to review annual technical reports of mature projects (defined as distributing ivermectin to endemic communities for seven or more years) on behalf of the TCC, and to support in-country operational research agenda in relation with CDTi implementation. An external evaluation system was established since the inception of the Programme. Evaluations are undertaken every five years, supported financially by the donor community and organized by the CSA. The evaluation teams are composed of scientists with a relevant background in public health. Three mid-term external evaluations have been undertaken in 2000, 2005, and 2010. They have made recommendations that allowed the JAF to make decisions on the mandate and future of APOC [20–22]. The next evaluation is planned for 2015.

## The Way Forward

The structure and management framework of APOC was determined based on the OCP experience. This mechanism has demonstrated efficacy in achieving APOC’s initial goal of establishing sustainable community-directed systems for ivermectin distribution that effectively controls onchocerciasis as a public health problem. In addition, the CTDi and CDI strategies have significantly contributed to scaling up other health interventions, such as control of lymphatic filariasis, distribution of insecticide-treated bed nets, and vitamin A supplementation, among others [20]. The success of APOC has prompted the JAF to extend the Programme beyond 2015, support countries in achieving elimination of onchocerciasis, use acquired expertise to benefit other targeted NTDs amenable to the PC strategy, and strengthen health systems at the community level across Africa. The evolution of APOC after 2015 is captured in the development and adoption of a concept note document and indicative budget to transform APOC into a new regional entity, provisionally named Programme for the Elimination of Neglected Diseases in Africa (PENDA) [23,24]. This new entity will have a mandate for “the coordination of the implementation of the elimination of onchocerciasis and lymphatic filariasis, and support interventions for other PC-NTDs in Africa” [24].

**Fig 2:**
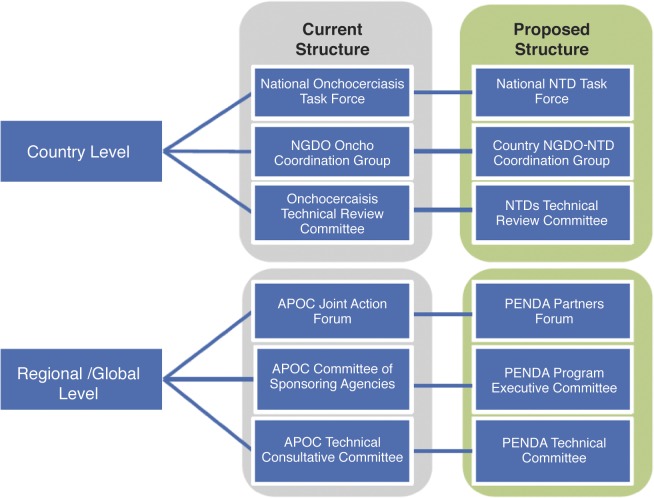
Possible evolution of APOC structures and mechanisms.

The global momentum and commitment for the elimination of targeted NTDs [25,26] requires putting in place adequate collaboration mechanisms and structures at all levels to ensure effectiveness, efficiency, and synergy of interventions. In this environment, APOC structures and management framework may evolve towards complementarity with other NTDs programs in PENDA. When APOC mechanisms have a competitive advantage, they should be extended to serve other NTDs in PENDA, for example, with the APOC Trust Fund and the JAF. At the same time, PENDA should also benefit from the added value of proven approaches for particular situations such as conflict, post conflict, urban settings, and problematic areas. With respect to global and regional governance and management structures, due attention should be given to the structure and management of NTD control and elimination programs at the national level, in accordance with country NTD master plans. Thus, the NOTF should be extended to cover other NTDs. Fig 2 depicts a possible evolution of APOC structures and mechanisms. Such an evolution would require the revision of the Memorandum of APOC [9]. The APOC Secretariat will support the transition of APOC from a single disease entity to a regional NTD elimination program and help ensure that future generations in Africa live free from the threat of debilitating and preventable diseases.
